# “A Double Decline in Character”: A Grounded Theory Analysis of Female Drug User Experiences in Iran

**DOI:** 10.1177/00914509241312578

**Published:** 2025-01-17

**Authors:** Alireza Momeni, Matilda Hellman, Maziyar Ghiabi

**Affiliations:** 1 Department of Social Sciences, University of Eastern Finland, Kuopio, Finland; 2 Department of Sociology, Uppsala University, Uppsala, Sweden; 3 Faculty of Social Sciences, University of Helsinki, Helsinki, Finland; 4 Faculty of Humanities and Social Sciences, University of Exeter, Exeter, UK

**Keywords:** addiction, drug users, substance abuse, grounded theory, stigma, women

## Abstract

Using a constructivist grounded theory design, this study explores the perception and experience of Iranian women living with drug use, identifying everyday conflicts and coping strategies that enable them to manage their situation. We conducted unstructured, in-depth interviews with abstinent drug users (11) and healthcare professionals (2) at three rehab centers in Tehran, Iran. In line with our grounded theory aspirations to explore the social meanings of women’s drug use and addiction, we supplemented the interviews with a surrounding material consisting of articles published in the main national newspaper from 2015 to 2018 and a film documentary (10 parts, 3 h) entitled “Iranian Women of Addiction,” (*Shab boohay-e-sokhteh*) (2010–2011). Our analysis resulted in two main bundles of meaning-making that we claim are permeating the everyday lives of Iranian women with drug use: the double decline in character, and self-shielding. The study reveals the simultaneous presence of two stigmatized identities: drug use and sex work (*fahsha*). It shows that the stigma of sex work is closely connected to a drug-related identity, irrespective of whether women are actively involved in sex work or not. The findings illustrate how societal views are biased against drug user women in the domain of drug use, diminishing their presence and voice. The interviewees tried to manage by developing a peer network, adopting a protective role, and becoming intrinsically motivated to quit drug use. The study furthers our understanding of women’s alarming and complex realities in traditional Islamic and familistic patriarchal structures.

## Introduction

According to the United Nations Office on Drugs and Crime (UNODC), some 3 million Iranians (among nearly 85 million inhabitants) are estimated to use illegal drugs regularly. Of these, more than 90% are believed to be male. However, Iranian health authorities have repeatedly noted that rates of substance use and dependency are on the rise among Iranian women, even if it has not shown up in the public statistics (Roshanfekr et al., 2019). Over their lifetimes, 1.5% of Iranian women (15–64 years) have met the criteria for drug use (UNODC, 2022). Research on gendered aspects of drug use and addiction points out that women may be rendered “invisible” as masculinity aspects dominate drug cultures (Hajiha et al., 2018); this is further exacerbated by the ethical conundrum and political liability that female drug use and addiction have represented for the Islamic Republic, making public authorities reticent to acknowledge women’s changing drug use patterns (Anaraki, 2022; Christensen, 2011; Ghiabi, 2019a). The dominance of male experiences is especially firm in ultra-patriarchal polities such as Iran (Etebari, 2014; Mokri, 2002). Thus, female drug users run significant risks of becoming marginalized and disempowered through stigma, invisibility, and misrepresentation (Purdie et al., 2008; Warner, 2008), and they tend to represent a socio-economically more disadvantaged group than their male counterparts (Chang, 2020; Moradinazar et al., 2020). This directly affects women’s access to treatment, welfare, and care services.

In the past, the domination of male populations and male experiences in addiction research (McHugh et al., 2018; Metz & Fischer, 2010) has led to generalized conclusions about female experiences (Metz & Fischer, 2010). It was not until the 1980s that the differences between male and female drug use started to receive more specific scholarly attention (Bepko, 2014; Rosenbaum, 1981; Rosenbaum & Murphy, 1990). When it comes to drug use in Iran, there is now significant literature on Iranian men’s substance use problems (e.g., Aghakhani et al., 2017; Amini-Rarani et al., 2020) and some about their spouses (Fereidouni et al., 2015; Joolaee et al., 2014; Panaghi et al., 2016). However, the drug use of Iranian women is a neglected area in qualitative studies. We have a limited understanding of how drug use and addiction manifest in this population and, also more generally, in patriarchal Muslim cultures and religious polities. women drug users comprise a vulnerable population.

Women’s substance use and addiction problems are known to be multi-layered, often perceived as devalued and “objectified” within societal norms and values, leading to repression (Hellman & Rantala, 2012).

This grounded theory (GT) study investigates the workings of such mechanisms and forces in an Iranian context. The primary data consists of several in-depth interviews with eleven female informants who have experience of drug problems. We have also interviewed two social workers with several years of experience in fieldwork with women drug users in Iran. In addition to the core material with direct insights into the lives of female drug users, we analyzed broad secondary data consisting of documentary films and media texts. In accordance with our GT-ambitions, this additional secondary data has served as contextualizing support for the excavation of mechanisms that lead to a distilling of the main findings (the “theorizing” of GT, see Charmaz, 1995; Glaser, 2005).

The objective of the study is to uncover some main traits of logic that permeate the “sociocultural integrated existence” of women drug users in the Iranian context (Stallwitz, 2012; Robertson, 2016). By approaching drug users’ existence as socioculturally integrated with the world around them, we aim to capture how the rigorously controlling moral, legal, religious, and misogynist codes and norms of an Iranian drug-using context show up in the study data. We identify a “being-in-the-world” of perceptions, experiences, conflicts, and coping strategies that shed light on the roots of some stereotypes and the stigma women face daily.

## The Iranian Context

During the past century, Iran has grappled with drug-related issues (Ghiabi, 2019a), having the highest global rate of opioid addiction, with around 4 million users, or 5.5% of the population (UNODC, 2008). There are several reasons why Iranians continue to use opium as their preferred opiate (Ghiabi, 2019a; UNODC, 2010), including medicinal purposes, geographical location, and “the ex-post facto consequences of the Iran-Iraq War” (Momeni, 2021, pp. 28–29). Research shows that Iranian women who are drug users and are living in the centers of the Welfare Organization (*Sazman-e-Behzisti*) predominantly use opium and opium residues (*Shireh*) (Roshanfekr et al., 2019). However, a shift to or combination with synthetic substances is now occurring, especially among women (Ghiabi, 2019a; Sarrami et al., 2013). Iranian drug policy has shifted from medical control under the Pahlavi monarchy to total prohibition post the Islamic Revolution in 1979, when drug use was criminalized under prohibition laws (Etebari, 2014; Ghiabi, 2019a; Madani et al., 2011). This means that possessing illegal drugs, also for personal recreational consumption, is a criminal offense that could lead to arrest and incarceration. Moreover, recidivism risks tougher sentences, including life and death penalty. Iran has topped the world’s ranking for the number of drug-related death sentences in 2022 (Giada et al., 2024), though since 2018, the country’s Judiciary has introduced a suspension of the application of the death sentence for non-armed drug offenses. Armed drug trafficking still carries the risk of a death sentence, especially in the southeast region of Sistan-Baluchistan, bordering Afghanistan and Pakistan. In practice, public authorities have demonstrated variable degrees of tolerance and flexibility, especially when it comes to substances perceived as carrying fewer risks for public health, such as cannabis and opium. In the country’s major cities, there has also been a pragmatic approach toward the public display of drug consumption. This is often reported in national newspapers with reference to large gatherings of homeless people using drugs in parks, under bridges, and along highway routes without systematic police repression (Ghiabi, 2020). Research suggest that the number of women using drugs in public parks and public spaces has increased, a fact reinforced by the increasing number of drug-related offenses attributed to women since the 2000s (Ghiabi, 2019b). Different legal and moral scripts regarding gender, class, and space coexist in the not-very-consistent control policies in major urban areas (Ghiabi, 2019b, 2021).

Since the mid-2000s, the Iranian state has introduced nationwide harm reduction and public health-oriented policies (Ekhtiari et al., 2020; Ghiabi, 2019a). This initially occurred in response to a hidden epidemic of HIV/AIDS that spread from drug offenders in prisons, often through female spouses to the general population, especially in the Western regions of Iran (Behrouzan, 2010), where the infrastructural and human toll of the 8-year war with Iraq had left a more profound mark (Alavi et al., 2021; Ghiabi, 2018a, 2019a; Himmich & Madani, 2016; Nissaramanesh et al., 2005).

Iranian authorities use gendered viewpoints and terminology when describing and treating drug-dependent women. These viewpoints are reflected in the officials’ plans and proposals for managing women with substance use problems. The perspectives of the affected women themselves are conspicuously absent from these plans, proposals, and policies. Systemic legal discrimination against women in many Muslim societies can be traced back to state codes rooted in Islamic jurisprudence, specifically family laws (Mir-Hosseini et al., 2014). Plans include “collecting women addicts from the streets” as so-called “risky” drug (ab)users (Ghiabi, 2018b) and “compulsory treatment” (Hamshahri, 2016; P5Q64-6; P3Q22), both to be implemented according to the law (article number 16), (Ghiabi, 2018b; Moghanibashi-Mansourieh et al., 2017). The most controversial proposal to date is the “sterilization of addicted homeless women” (Hamshahri, 2017; BBC Persian, 2016). The rationale behind this initiative was to prevent a greater threat to the population brought on by drug use homeless women who would end up in sex work. Consequently, it was reasoned that many unwanted children would be born without secure and reliable guardians and then be sold, creating a vicious circle of social harm and promiscuity. Sterilization of homeless women was thought to break this vicious cycle (Hamshahri, 2017). The proposal gave rise to great controversy, and ultimately, the government did not approve it. However, this type of proposal is part and parcel of the wider framework of the intractability of drug offenders in Iranian public opinion (despite or because of the high number of drug users). Public opinion has been condemning and suggesting that the death penalty for all drug users is not rare. This is an outcome of a long tradition of scapegoating drug use(rs) for the country’s social and political shortcomings (Ghiabi, 2019a).

## Materials and Methods

### Sampling and Recruitment

We applied purposive sampling to access a wide range of users’ experiences in Iranian women’s substance use circumstances. The interviews were conducted in three primary settings: two outpatient rehabilitation clinics and a drop-in center (DIC) in Tehran, Iran. Additional research participants were recruited from a DIC for women in the Shush area south of Tehran. The center offers harm reduction services (free needles, free condoms, free meals, and social work and counseling services) for women who struggle with drug use problems and/ or are sex workers. The DICs are also reference points for the broader neighbourhood in receiving health consultation and welfare support in ad hoc instances.

We selected participants indirectly through the service provider based on their shared experience or knowledge of living with addiction. After we had reached a range of experiences through our first sampling strategy, we utilized a theoretical sampling based on the emergent codes, concepts, and categories from the progressing data collection and analyses (Strauss & Corbin, 1998). At this stage, we asked additional questions during the interviews, seeking out participants with a broader diversity and background and expanding the research setting. We are aware of the potential biases and limitations inherent in this approach and have taken steps to address them through triangulation and transparency. We continued this procedure until a theoretical saturation was reached (Glaser & Strauss, 1967; Strauss & Corbin, 1998).

### Design

Constructivist GT strategies employ an inductive approach, progressing through analytical rounds to develop a theoretical understanding of the studied phenomenon, in this case, the sociocultural engrained experience of female drug users and sex workers in Iran. The main idea is that the researcher moves thoroughly and systematically between and within the materials in search of segments or bundles of meanings and logical meaning-making. Gradually, an understanding of the main ingredients in the studied complex sociocultural existences is formed. Various types of material are often used together as sources for the general conclusions that are drawn. These general conclusions regarding the main logic and mechanisms at play are the theorizing in GT. The methodology is ideal for topics with limited prior knowledge (Artinian et al., 2009 Momeni, 2021; Robson, 2002). Constructivist grounded theory (CGT) is valuable for studying silenced and marginalized experiences not addressed in existing sociological categories (Charmaz, 2014a; Mills et al., 2006). Our analysis began with the fieldwork team members identifying major categories and examining participants’ intersections for forming identity features (e.g., Bowleg, 2008; Warner, 2008). The study works with four data sets: two primary materials, including in-depth key informant interviews with drug users (data set 1) and social workers (data set 2). In addition, secondary data consisting of a ten-part documentary (data set 3) and 24 newspaper articles (data set 4) were collected and analyzed for the study.

The primary field researcher collected all data sets. This person conducted 11 months of fieldwork and data collection. Interviews were collected over two months (June 10 to July 20, 2015), followed by two supplementary stages: reviewing a 10-part documentary (September 2015 to May 2016) and analyzing 24 newspaper articles (January & February 2018). This researcher has nine years of experience as an occupational therapist working with individuals with mental illness or substance abuse disorders in Iran and has also developed an original GT on male addiction rehab clients in Iran (see Momeni, 2021). This insider perspective provided invaluable local knowledge for understanding the study context. Field notes, reflections, and analytical memos added depth (Jacelon & O’Dell, 2005; Lincoln & Guba, 1985).

### Interviews

The study participants with their own drug use experience (data set 1) were 11 women between 22 and 53 diagnosed with substance abuse disorder by a psychiatrist. At the time of the interviews, the interviewees were receiving treatment during an abstinence phase at the rehabilitation institute in Tehran, Iran. None of them reported any involvement in sex work, even though this topic came up as one of the main traits in the interviews’ meaning-making. The demographic characteristics of interviewees are presented in Table 1.

The two additional interviewees (data set 2) were social workers aged 62 and 63 who worked as healthcare professionals at the centers and had seven years of experience at the DIC center. Conducting those interviews alongside the main participants validated preliminary information and provided a holistic understanding of the phenomenon. We also considered both the frontline experiences of drug users and the organizational dynamics that shape those experiences, facilitating comprehensive analysis.

All face-to-face interviews lasted between 30 and 60 min except for three interviews that lasted over an hour. In total, 29 meeting sessions were conducted, and some interviewees were interviewed multiple times. Around 10 h (9 h and 46 min) of audio recordings and over 200 pages of transcriptions were obtained from the interviews. At the start of each interview, we sought permission from participants to record supplementary information and keywords. We applied “debriefing” strategies by taking notes on keywords during interviews to summarize discussions and obtain participant feedback (Kvale, 1996, p. 127). All interviews were audio-recorded and transcribed. The primary field researcher audio-recorded and transcribed the materials and then worked with a translator with a bachelor’s degree in English literature to translate them.

### Secondary Data

Additional secondary data materials included a documentary film (data set 3) and newspaper articles (data set 4), allowing us to assess results in a contextualized and socioculturally sensitive way (Glaser, 2005, p. 141; Patton, 1999). We reviewed a 3 h documentary, “Iranian Women of Addiction” (2011) (*Shab boohay-e-sokhteh*), released on YouTube in the year 2011 (Akbari, 2011). The documentary provided personal stories and expert interviews, offering firsthand, yet of course edited, perspectives on drug use. This aligns with the trend in Iranian media of exploring drug addiction themes in films and TV shows.

Data set 4 consists of 24 articles about women’s addictions from major national newspapers (Hamshahri, Iran, and Etemad) from 2015 to 2018. Iranian newspapers often report direct quotes from political agents, experts, civil society groups, and government representatives, allowing us to form an understanding of the Iranian public discourse on women and diverse drug phenomena. Based on our primary data sets, we conducted a full content analysis and deconstruction of media and public discourse. However, due to space limitations or emphasis, this paper will focus on our primary data material. Occasionally, we reinforce and illuminate our observations in the light of our secondary data, enhancing the depth and enriching our understanding of the phenomenon (Patton, 1999).

### Analytical Proceeding

In line with CGT protocols, our data analysis entailed open coding and began concurrently with and after the initial interviews. Each interview was transcribed verbatim and coded, following a repetitive process for subsequent interviews. This approach allowed us to theorize and refine our study progressively. Over time, we deepened our grasp of interview content by carefully reviewing and transcribing tape recordings, enhancing our theoretical sensitivity to the data. This iterative process enabled us to uncover significant meanings possibly overlooked during interviews, informing subsequent sessions.

Analyzing diverse data, including interviews and documentaries (i.e., documentary films and newspapers), involved a systematic approach to extracting meaningful insights. We iteratively read and assigned codes to segments with similar content or meaning. This reading, coding, and re-coding process enabled us to identify “segments”—distinct data sections extracted for closer examination and interpretation. Second, we converted all information from the documentary film into text format. The documentary included personal stories and interviews with experts, which formed a backdrop for both interviewed subjects’ situations and how drug stories are narrated to the public in the Iranian media. The documentary film obscured drug users’ faces to maintain confidentiality, placing more emphasis on spoken content than visuals. This feature allowed for transcription and analysis of stories and interviews within the film, focusing on spoken content rather than visual cues. We watched the film multiple times to familiarize ourselves with the interviewees’ voices and the contextual aspects of the interviews. This facilitated identifying and extracting portions that pertained to our research question. By transcribing the highlighted portions of the film, we converted them into segments and coded them for subsequent comparison with the initial codes and patterns identified while analyzing the preliminary interviews. By integrating diverse qualitative data and including various perspectives, we successfully utilized a “constant comparative strategy” (Glaser & Strauss, 1967) to cross-check all codes and categories and achieve triangulation (Patton, 1999). The data were analyzed with the technical aid of ATLAS.ti software. We separated, sorted, and compared segments (Charmaz, 2006) to identify common patterns and insights to comprehensively grasp the research topic. We internally compared the different meaning-making of the materials and reflected on them given theoretical frameworks.

### Ethical Considerations

The Research Ethics Committee at the University of Eastern Finland granted ethical approval for the study in April 2015. Special attention was given to the vulnerability and possible trauma of the study participants and gender-related, academic, and geographical aspects of consideration regarding the interaction between interviewer and interviewee. Before the interview, participants were given a short introduction about the study’s purpose and procedures, and informed consent was verified. The participants’ names and identities were not disclosed in the study’s collection, analysis, or report. Additionally, transcripts and audiotapes of the interviews were stored in a secure location throughout the research period. The aim was to ensure no risks or inconveniences were involved in participating in the study throughout our procedures.

## Findings

Segments and meaning-making bundles were merged into two main categories of central sense-making. Each category sheds light on a specific aspect of the research participants’ experience (see Table 2). These categories emerged from 909 initial and 79 focused codes and have been developed (“distilled”) in the project team through conversations during the research process. The categories are double decline in character and self-shielding. They all entail paths of “objectification” through in-between positions of subjects and objects (Kristeva, 2024). Their statuses, including internalized self-identity and societal position, can be influenced by the dominant Iranian cultural and social norms surrounding body image, health, female roles, shame, stigma, and guilt.

The study participants’ stories convey the negative perceptions and social rejection they face, as drug use and addiction are seen as deeply discrediting attributes in society. This theme is conceptualized through the lens of stigma. The participants’ accounts highlight various aspects of coping with stigma, illustrating the daily experiences of facing social exclusion, rejection, and discrimination. The individuals responsible included relatives, friends, male bosses, and healthcare professionals.

In the following sections, we will highlight excerpts from the interviews to convey the essence of each category. This will demonstrate how the subjects navigate their lived reality. For each citation in the analysis, we have renamed each participant (P) with a number, and for each Quotation (Q), the order in which the citation has been coded in the software.

### The Double Decline in Character

“The double decline in character” refers to a situation in which female drug users encounter double stigma due to drug and sex work, regardless of whether they engage in sex work. The interviews entailed prominent themes of dehumanization, objectification, and double stigma. Participants encountered double stigma, rooted in deeply ingrained gender biases in societal and familial attitudes toward drug use and addiction. A participant shared:

For our family, a woman’s addiction is considered very bad; they consider it very bad that she becomes an addict. Now, about a man, they say he is a man. My mom says if he is an addict, he is a man, don’t care, no matter what happens, even if he doesn’t have money, well, finally, somehow, he will make his money, but you are a woman, the first thing that you are doing, I apologize for saying that, is that you are going to sell yourself. (P3: Q58)

The participant’s frequent apology for openly discussing sex work reflects her internalized stigma and shame, arising from the assumptions that women who use drugs are also likely to engage in sex work. The same participant noted:

Imagine, I used drugs so that I didn’t need any man; if I had money, I wouldn’t even look at a man. But again, their look, their look, whenever they look at me, for example, when they find out that I have an addiction, they look differently; I apologize for saying that […] Someone says she is a promiscuous [Harze: ﻫﺮﺯﻩ], […] and says, look how many men she is sleeping under. (P3: Q44)

One participant describes feeling trapped by societal labels that associate drug use with sex work and shared her experience with potential objectification and sexual abuse.

They [public] think that a woman who has taken drugs can definitely do something; for example, she can easily allow them to have sex with her. […] I experienced this. […] It’s not really a reason because I used drugs for 12 years, but I didn’t have anything like this [sex work]. For example, I used to change my place of work once every three months, and I couldn’t stay at one job anymore because they all looked at me abusively [Nazar Dashtan:نظر داشتن] at my workplace. (P4: Q25-Q39)

One of the social workers points out that women’s addiction is often “synonym to sex work.” (P12: Q26). According to another participant:

As soon as this woman is identified as an addict, a series of stigmas and labels begin; they say she is a prostitute, she may have AIDS, she may have syphilis, gonorrhea, a thief, and a criminal. Yes, all add up, and her human identity and rights are violated. (P13: Q58)

### Self-Shielding

Self-shielding pertains to the strategies employed by interviewed female drug users to protect themselves from danger, risk, or unpleasant experiences. These strategies include developing a peer network, adopting a protective role, and becoming intrinsically motivated to quit drugs.

### Developing a Peer Network

Despite the challenges faced by women who use drugs, the participants’ stories revealed that they are not helpless individuals who passively accept the situation without striving to change it. For example, they developed a robust support network to understand and support them without judgment. A participant shared:

They [the public] don’t accept an addicted person anywhere. They don’t accept me anywhere. For example, if I try to find a job, it’s not easy. It was very difficult, but I found a place where we all empathized. They all empathize with me, and I can show them love and help them a lot, and I can learn a lot from them. (P4: Q20)

A participant shared how these support systems contribute to finding intrinsic motivation for recovery and maintaining sobriety:

Sometimes, I mess up; I have a craving [to use drugs]. Well, I do some things; for example, I attend meetings, reach out to a friend who understands what I’m going through or a guide, and go to a class. (P10: Q11)

### Adopting a Protective Role

The participants reported various strategies to protect themselves from potential sexual abuse in their daily interactions, such as when seeking drugs. These range from resistance and aggression (P4: Q28) to implementing deceptive protective strategies. A participant pretending to be pregnant demonstrates a proactive and adaptive response to possible sexual abuse:

I had my own trick [to handle the situation]. I lied; for example, I pretended that I was pregnant. I covered my belly. So that the [abusive] view wouldn’t exist anymore. (P10: Q29, 31 and 32)

### Becoming Intrinsically Motivated to Quit Drugs

While participants’ accounts contain personal and social challenges due to drug use, they may find a sense of empowerment within these constraints, serving as powerful motivators to change their situation. A participant noted:

I was tired [of the drug-related circumstances], and it was hard financially. It was difficult socially; I couldn’t go anywhere. For example, I couldn’t go to my friend’s house or a party at my mother’s; this [situation] motivated me to quit. (P11: Q24)

## Discussion

This study has shed light on some ways in which gender-based constraints and drug-related stigma, as well as sex work stigma, intersect to undermine the self-worth and identity of female drug users in Iran. Despite facing challenges, these women demonstrate strength and resilience in overcoming their circumstances, finding ways to cope, and even thriving against the odds. Our research emphasizes the need for interventions and support systems tailored to address these women’s unique challenges. We discuss our findings about existing literature to illuminate this complex issue and highlight the identified gaps.

The study suggests that the intersection of drug use and gender creates a complicated condition in which two forms of stigma profoundly shape identity. Our findings show that the label of sex work is inextricably linked to a drug-related identity, regardless of whether women are actively engaged in sex work or not. Hence, the double decline in character arising from the intertwined realms of drug use and sex work stigma is the product of deeply ingrained gendered logic. In this sense, the issue we are dealing with is not just addiction or “drug use” but a gendered, judgmental, and misogynistic attitude—a culture of judgment—that leads to the harm associated with drugs. Our findings support other studies, highlighting existing stereotypes related to promiscuity and sex work for female drug users (Meyers et al., 2021). Studies in the Iranian context have established a significant link between drug addiction and sex work, with drug use often preceding or following engagement in sex work (see Roshanfekr et al., 2019). However, these studies often lack the depth of exploration regarding how this association affects women’s daily existence.

The coexistence of two stigmas resulting from two forbidden acts (drug use and sex work) leads women to become objectified and abused. In Iran, drug use has been criminalized under prohibition laws since 1979. Sex work is illegal and severely punished under Islamic law in this context. However, Islamic jurisprudence permits “temporary marriages,” known as sigheh (or mut’a in Arabic); marriage is a contractual agreement rather than a sacrament in Islam. Here, two parties can contract a marriage for a specific period based on mutually agreed terms, including monetary or dowry payment. This has resulted in the instrumental adoption of temporary marriages for sex work in a legal framework recognized by religious jurisprudence (see Haeri, 2014; Yaghoobi, 2020). This situation has not affected the negative connotation of women perceived as sex workers, even when acting in legal or formal frameworks. Hence, a negative moral burden and devastating consequences arise by assigning a sex worker’s identity to somebody.

Judgmental attitudes often reduce women to mere representations of these stigmas, making it challenging for them to shake off these stigmatized identities. Based on our analysis, this situation often puts women who use drugs at greater risk of objectification and exploitation. These findings are consistent with Covington’s study (1997). While she does not explicitly discuss the existence of double stigma among women, she points out that "the stigma against addicted women is often expressed in sexual terms" (Covington, 1997, p. 3). Our findings support the findings of intersectionality research that highlight how overlapping stigmatized identities can magnify social exclusion (Bowleg, 2008).

Participants’ narratives reveal some strategies for coping with both drug-related issues and the double stigma rooted in a misogynistic culture. These strategies are based on accepting an active role in their drug-related condition, where women are likely to be open and responsive to outside help and support others as well. Our analysis indicates that participants try to develop a sense of resilience that drives them to overcome drug-related challenges and improve their lives. For example, experiencing rejection and sexual abuse due to double stigma may drive them to seek supportive communities or get involved in advocacy work to contribute to society. While numerous studies emphasize the importance of support networks, limited research exists on the specific strategies women drug users employ to build and sustain these networks (Lee & Boeri, 2017; Moos, 2007). Additionally, few studies explore the long-term impact of these support systems on women’s resilience and recovery. They may find intrinsic motivation to change and actively work to manage their challenges rather than denying or avoiding them. This intrinsic motivation is crucial for sustaining long-term recovery and fostering a positive self-identity despite the pervasive societal judgment they face.

This study contributes to intersectionality theory by highlighting how multiple stigmas intersect to exacerbate women’s objectification. It underscores the need for theoretical frameworks that account for the complex interplay of multiple stigmatized identities.

We suggest an ethical shift focused on "harm reduction" to prioritize compassion and well-being over punitive measures. This shift aims to fundamentally change public attitudes toward drug use and women. Our primary goal is to implement a women’s empowerment approach, establishing a foundation for long-term resilience and self-sufficiency.

## Limitations and Future Direction

This study has referred to the notion of “women drug users” in a general sense. A distinction between the types of drugs being used, the extent of substance use, and the consequences of substance use are beyond the scope of this study. The women’s experiences in the current study represent a small minority. It is important to remember that Iranian women and Iranian women drug users do not form a homogeneous group; socioeconomic class, cultural and ethnic background, and environmental setting affect their relation to the experience of addiction. Our study focuses primarily on the urban population of Tehran. Geographic location and spatial distribution also impact the nature of problems and access to help structures. Interviews with Iranian women from other regions are, however, likely to reflect the same types of patriarchal norms and stereotypes (Kian, 2011), though with variations of intensity and forms (e.g., in the Caspian region with more relaxed gender codes or in the South-East with stricter cultural norms).

## Conclusion

The findings of this study highlight the challenges experienced by women with drug-related issues in patriarchal cultures with drug control systems that are designed to punish and exclude women who use drugs. It emphasizes that despite their challenges, these women show strength and resilience in overcoming their circumstances, coping, and even thriving against the odds. The study also emphasizes the need for interventions that consider the gender-specific aspects of drug use within the cultural and policy context, empowering women to handle their constraints effectively. Furthermore, this study is significant in addressing the issue of drug use and addiction in Muslim-majority countries, where there has been minimal scholarly attention, and it calls for detailed empirical research in this area.

## Figures and Tables

**Table 1:** Characteristics and Background Information of the Women Drug Users Participating in the Interview Study (*N* = 11).

Participants	Characteristics	*N*
Age	22–37	9
	38–53	2
Marital status	Married	4
	Widow	7
Educational degrees	BSc	1
	Diploma	5
	Under diploma	5
Occupation	Employed	2
	Unemployed	5
	Stay-at-home mom	4
Total		11

**Table 2:** The Main Categories, Descriptions, and Sub-Categories.

The main categories	Subcategories	Description
The double decline in character	Drug-related identity Drug-related Stigma Whore stigma Broken soul Damaged self Misrecognition	Illustrates participants’ perceptions of how sociocultural structures and processes treat them and the impact of these structures on their own sense of self and identity. Here, the objectifying elements are heavily gendered, pertaining, for example, to assumptions regarding drug use as automatically being associated with promiscuity, sexual availability, and sex work.
Self-shielding	Secret identity Developing a peer network Adopting a protective role Motivation to quit	Pertains to coping strategies that the participants employ to protect themselves from unpleasant or stigmatizing drug-related circumstances.
